# Collective Cognition in Humans: Groups Outperform Their Best Members in a Sentence Reconstruction Task

**DOI:** 10.1371/journal.pone.0077943

**Published:** 2013-10-17

**Authors:** Romain J. G. Clément, Stefan Krause, Nikolaus von Engelhardt, Jolyon J. Faria, Jens Krause, Ralf H. J. M. Kurvers

**Affiliations:** 1 Department of Biology and Ecology of Fishes, Leibniz-Institute of Freshwater Ecology and Inland Fisheries, Berlin, Germany; 2 Department of Crop and Animal Sciences, Humboldt-University of Berlin, Berlin, Germany; 3 Department of Electrical Engineering and Computer Science, Lübeck University of Applied Sciences, Lübeck, Germany; 4 Department of Animal Behaviour, University of Bielefeld, Bielefeld, Germany; 5 Department of Ecology and Evolutionary Biology, Princeton University, Princeton, New Jersey, United States of America; Cajal Institute, Consejo Superior de Investigaciones Científicas, Spain

## Abstract

Group-living is widespread among animals and one of the major advantages of group-living is the ability of groups to solve cognitive problems that exceed individual ability. Humans also make use of collective cognition and have simultaneously developed a highly complex language to exchange information. Here we investigated collective cognition of human groups regarding language use in a realistic situation. Individuals listened to a public announcement and had to reconstruct the sentence alone or in groups. This situation is often encountered by humans, for instance at train stations or airports. Using recent developments in machine speech recognition, we analysed how well individuals and groups reconstructed the sentences from a syntactic (i.e., the number of errors) and semantic (i.e., the quality of the retrieved information) perspective. We show that groups perform better both on a syntactic and semantic level than even their best members. Groups made fewer errors and were able to retrieve more information when reconstructing the sentences, outcompeting even their best group members. Our study takes collective cognition studies to the more complex level of language use in humans.

## Introduction

Group-living is widespread among animals and one of the major advantages of group-living is the ability of groups to solve cognitive problems that exceed individual ability [1-6]. This process is known as the many wrongs principle [7], swarm intelligence [1,5], wisdom of crowds [8] or collective cognition (CC) [3]. Fish, for example, make faster and more accurate decisions in groups than when alone [9], in ants larger colonies are faster at finding the best nesting sites [10] and in birds larger groups are more successful in innovative problem solving [11]. Also humans can make use of CC and CC has been shown to solve a number of different problems including predicting the results of elections [12,13] solving letters-to-numbers problems [14,15] and increasing speed and accuracy at reaching a target when navigating as a group [16]. 

 A remarkable feature of humans is the use of a highly complex language. Language is thought to have played a critical role in the evolution of hominids [17] giving them a unique way of sharing information among conspecifics. Moreover, group discussion is still the most widely used method by human groups to arrive at consensus decisions. Several studies have investigated CC of human groups with regards to quantity estimations and letters-to number problems (see 5 for a review). However, few studies made language itself the focus of their investigation. Here we simulated a realistic scenario to investigate the potential of CC in human verbal communication: individuals listened to a public announcement and had to reconstruct the announcement alone or in groups. This situation is frequently encountered by humans in their daily life, for instance at train stations or airports.

Communication analysis is challenging but recent developments of sophisticated methods in machine speech recognition have provided us with powerful tools that allow the analysis of syntax and semantics of human language [18]. Here we apply these novel tools to study if human groups can decrease error rate (syntax) and increase semantic understanding compared to single individuals in an everyday task. In this study we particularly focused on the question whether groups can outperform their best member.

## Material and Methods

### Experimental setup

We recruited 167 student volunteers from the University of Bielefeld (Germany) participating in a course on behavioural ecology (April 2011). Participants were divided in 21 groups. All groups consisted of eight members, except one group which had 7 members. Informed consent was obtained from all participants and data collection was anonymous. Prior to the experiment we communicated to all participants that they were allowed to leave at any time. All procedures were carried out in accordance with the Declaration of Helsinki. We deemed it unnecessary to apply for formal ethical approval for this study as it is highly unlikely that participants would feel uncomfortable in participating in this simple and straightforward task. Listening to a sentence and reconstructing a sentence is a very simple task that most people perform on a daily basis without any negative consequences. Moreover, the experiment was part of a student practical for which no ethical approval was required and the students used the data afterwards for learning about experimental design.

In the experiment, two sentences in German and of equal length were played back to the participants (See Table 1). These were announcements that are typically audible at a train station or an airport. We added echo, white noise and a 55 Hz tone to both sentences using Audacity (http://audacity.sourceforge.net) mimicking a real-life situation at a public place as for instance encountered at a train station or an airport.

**Table 1:** Overview of the sentences as used in this study.

**Train station announcement**	**Airport announcement**
Original sentence:	Original sentence:
Der Zug aus Reinfeld mit Weiterfahrt nach Hamburg-Dammtor, Abfahrt um 15 Uhr 32, fährt heute auf Gleis 19 ein.	Die Fluggäste des Fluges LG 327 nach Stettin werden gebeten, sich umgehend zum Flugsteig C 31 zu begeben.
Translated sentence:	Translated sentence:
The train from Reinfeld continuing to Hamburg-Dammtor, leaving at 15:32 arrives today on platform 19.	The passengers of flight LG 327 to Stettin are requested to go to gate C 31 immediately.
List of 8 semantic items:	List of 7 semantic items:
- the subject (train)	- the addressees (passengers)
- the origin of the train (from Reinfeld)	- the airline code (LG)
- the destination of the train (to Hamburg Dammtor)	- the flight number (327)
- the fact that the announcement is about a departure	- the destination of the flight (to Stettin)
- the time of departure (15:32)	- where to go (gate)
- the action of the train (arrives)	- the gate number (C 31)
- the date (today)	- the requested action (go to)
- the platform (platform 19)	

The original German sentences, the English translation of the sentences and the semantic ‘items’ of both sentences.

All groups underwent two treatments: “individual treatment” and “group treatment”. In both treatments, participants listened first to a sentence and were given 1 minute to individually write down the sentence as they heard it. Then, for the individual treatment, participants were permitted four additional minutes to improve their sentences individually. In the group treatment, the participants had four minutes to discuss and write down one consensual sentence. All groups received each sentence once (i.e., one sentence during the individual treatment and the other during the group treatment). The order of the two treatments (i.e., individual or group) and the two sentences were randomized so that each of the 4 combinations was performed with approximately the same number of groups (5 or 6).

The participants wrote their sentences on sheets that had 30 boxes and were asked to write one word per box and to leave blanks where they thought that a word was missing. The number of boxes far exceeded the actual number of words in the sentences to avoid limiting the participants or giving them a clue regarding the actual number of words.

### Analysis

The quality of the reconstructed sentences was evaluated on two levels: syntactic, i.e. regarding the correctness of the word sequences, and semantic, i.e. regarding the correctness of the pieces of information contained in the sentences. 

For the syntactic analysis we used the “Word Error Rate” (WER), which is the standard evaluation metric for speech recognition [18]. The WER is the minimum number of changes (insertions, deletions, and substitutions of words) needed to transform the correct sentence into the reconstructed one, divided by the number of words in the correct sentence (see Table 2 for an example). The WER was calculated using the Speech Recognition Scoring Toolkit (version 2.3.5) of the National Institute of Standards and Technology (http://www.nist.gov/itl/iad/mig/tools.cfm).

**Table 2:** Example for the computation of the Word Error Rate (WER).

The	train	to	London		is	delayed	for	fifteen	minutes	due	to	bad	weather
The	train	to	London	Euston	is	delayed	for	fifty	minutes	due	to		
				I				S				D	D

The first sentence is the correct one, the second sentence is the reconstructed one. The insertions, deletions, and substitutions are marked by ‘I’, ‘D’, and ‘S’, respectively. For this example the WER = (number of changes) / (number of words in the correct sentence) = 4/13.

The meaning of a sentence with a low WER is not necessarily more correct than that of a sentence with a high WER because the WER does not take the semantic relevance of words into account. Therefore, in our semantic analysis we looked at particular pieces of information, called “items” that constituted the meaning of the sentences. We identified 8 items for the train station announcement and 7 items for the airport announcement (See Table 1). We evaluated the semantic correctness using the following measures that are widely used in the fields of information retrieval and speech recognition [18].

Precision (P) = *Number of correct items in the reconstructed sentence / Total number of items in the reconstructed sentence*

Recall (R) *= Number of correct items in the reconstructed sentence / Total number of items in the correct sentence*

The precision measures the degree to which the retrieved information is correct. The recall measures how much of the available information was successfully retrieved. To evaluate the overall quality of information retrieval, it is common to combine them by computing their harmonic mean, called the F-measure [18]:

F = 2PR / (P+R)

The F-measure was calculated using an own script. We have added the code as supporting information (Analysis S1, S2).

### Decision mechanism

To understand how groups arrived at communal decisions we studied the transition from the collection of independent responses to the group response during the “group treatment”. For this we listed all eight independent responses per word per group and compared this to the group decision of that particular word and that particular group. We distinguished between the categories: consensus (i.e., all independent responses that were given were identical to the group response), majority (i.e., the group response corresponded to the word that was most often reconstructed during the independent responses), tie (i.e., the group response corresponded to one of two (or more) words that were most often reconstructed during the independent responses), minority (i.e., the group response was present in the independent responses but was not one of the words that were most often given in the independent responses) and invented (i.e., the group response was not present in the independent responses). We studied how frequently these different situations occurred and whether they led to better decisions. For this we calculated the rate of correct responses for the independent responses (varying between 0 and 1) and compared this to the group decision (either 0 or 1) (hereafter called: ‘success rate’). Whenever individuals had no answer for a particular word (i.e., did not hear it) we treated this as ‘incorrect’. 

 We also studied the group performance as compared to the combination of the best responses from all independent responses (i.e., combining the best answers of all given independent opinions). This allowed us to study if there was a so-called assembly bonus effect present which means that group performance is better than the performance of all individual group members or any combination of individual member efforts [19,20].

### Statistics

For the individual treatment, we calculated the WER and F-measure of all individuals and of the best individual after one minute and after four additional minutes. For the group treatment, we calculated the WER and F-measure of the best individual after 1 minute and the group performance (i.e., after four minutes of discussion). A direct comparison between the best individual after four additional minutes in the individual treatment and the group consensus after four minutes in the group treatment was not possible since we found strong effects of both treatment order and sentence (see below) preventing a direct comparison. Therefore, we ran a separate analysis within each treatment (i.e., individual and group) to quantify the effect of additional minutes on individual and group performance. The success of reconstructing the two sentences (measured by WER and F-measure) by (1) all members, (2) best members and (3) groups were analysed using (separate) generalized linear mixed models with a logit link function (glmmPQL function in package MASS in R, version 2.14.1). As fixed effects in all models we included time (i.e., 1 or 4 minutes) sentence and treatment order (i.e., first or second experiment). For the model including all members, we included individual nested in group as a random effect. For the models including best members or groups, we included group as random effect. 

## Results

### Individual treatment

**Figure 1:**
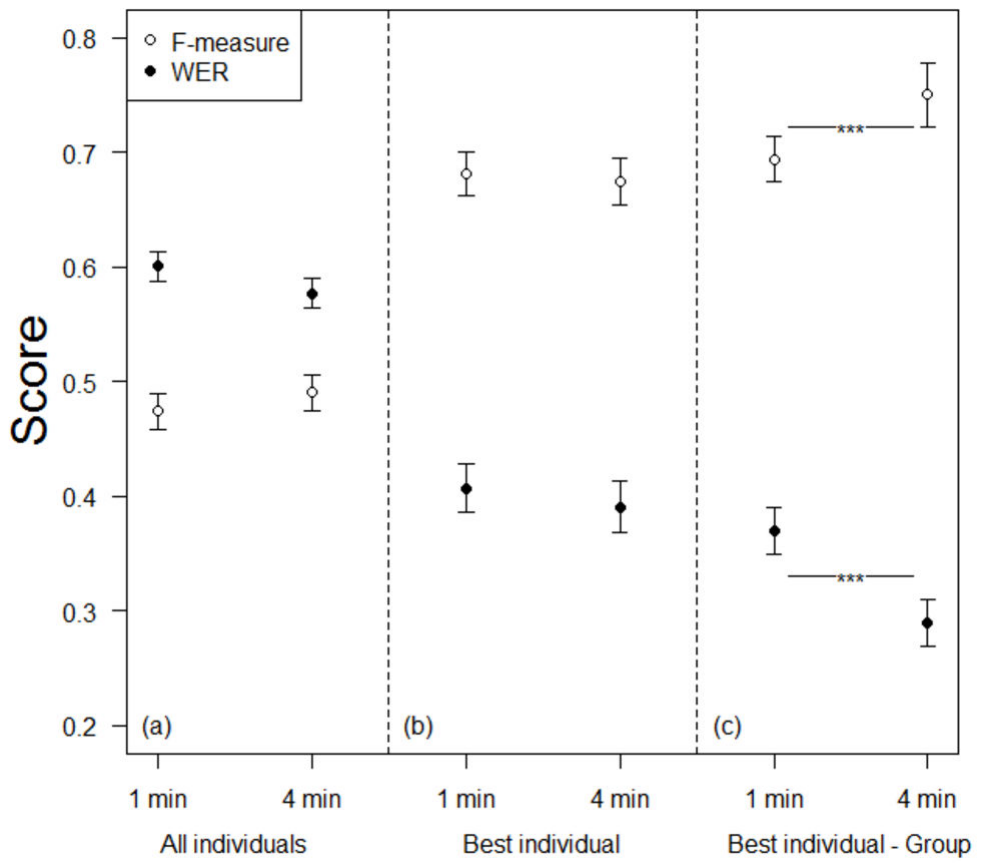
Groups outperformed their best members. During the ‘individual treatment’, (a) individuals did not improve their WER or F-measure with extra time. (b) Likewise, the best individuals of each group did not improve with extra time. During the ‘group treatment’, (c) groups had a lower WER and a higher F-measure than the best individuals. Shown are mean ± SE of WER (closed circles) and F-measure (open circles). Data are based on all sentences.

In the individual treatment, there was no difference in WER or F-measure after 1 or 4 minutes including all individuals (Figure 1a; Table 3). There was an effect of treatment order on WER and F-measure during the individual treatment (Table 3). Participants that started with the individual treatment performed worse during the individual treatment (i.e., higher WER and lower F-measure) than those that finished with the individual treatment. Additionally, there was an effect of sentence on F-measure but not on WER (Table 3). Likewise, the best individuals of the group did not improve in WER or F-measure with additional time (WER: 1 minute: (mean ± SD=) 0.41 ± 0.09, 4 minutes: 0.37 ± 0.10, *P* = 0.23; F-measure: 1 minute: 0.68 ± 0.09; 4 minutes: 0.67 ± 0.09, *P* = 0.45; Figure 1b).

**Table 3:** Result of the generalized linear mixed model analysis of the ‘individual treatment’ including all individuals.

**(*a*)Word error rate (WER)**				
	**estimate**	**Std. Error**	**t**	***P***
(Intercept)	0.858	0.113	7.597	< 0.001
Time	-0.100	0.066	-1.514	0.131
Order	-0.627	0.121	-5.167	< 0.001
Sentence	-0.254	0.121	-2.091	0.051
	**estimate**	**Std. Error**	**t**	***P***
(Intercept)	-0.737	0.120	-6.125	< 0.001
Time	0.070	0.076	0.918	0.359
Order	0.622	0.128	4.850	< 0.001
Sentence	0.626	0.128	4.874	< 0.001

(b) F-measure

Shown are the effects of time (i.e., performance after 1 minute or after 4 additional minutes), order (i.e., sentence being played first or second) and sentence (i.e., train station or airport announcement) on (a) the Word Error Rate and (b) the F-measure.

### Group treatment

Groups scored significantly better on both the WER and the F-measure than the best performing individual in the group treatment (WER: 1 minute: (mean ± SD=) 0.37 ± 0.09, 4 minutes: 0.29 ± 0.09; F-measure: 1 minute: 0.69 ± 0.09; 4 minutes: 0.75 ± 0.13; Table 4, Figure 1c). There was no effect of treatment order or sentence on WER or F-measure during the group treatment (Table 4). See also supporting information (Data S1).

**Table 4:** Results of the generalized linear mixed model analysis of the ‘group treatment’.

**(*a*) Word error rate (WER)**				
	**estimate**	**Std. Error**	**t**	***P***
(Intercept)	-0.754	0.145	-5.218	< 0.001
Time	-0.372	0.101	-3.670	0.002
Order	0.174	0.153	1.133	0.272
Sentence	0.254	0.154	1.655	0.115
	**estimate**	**Std. Error**	**t**	***P***
(Intercept)	1.087	0.201	5.399	< 0.001
Time	0.288	0.126	2.282	0.034
Order	-0.133	0.217	-0.614	0.547
Sentence	-0.341	0.217	-1.571	0.134

(b) F-measure

Shown are the effects of time (i.e., performance of the best individual after 1 minute or the group decision after 4 additional minutes), order (i.e., sentence being played first or last) and sentence (i.e., train station or airport announcement) on (a) the Word Error Rate and (b) the F-measure.

### Decision mechanism

**Figure 2:**
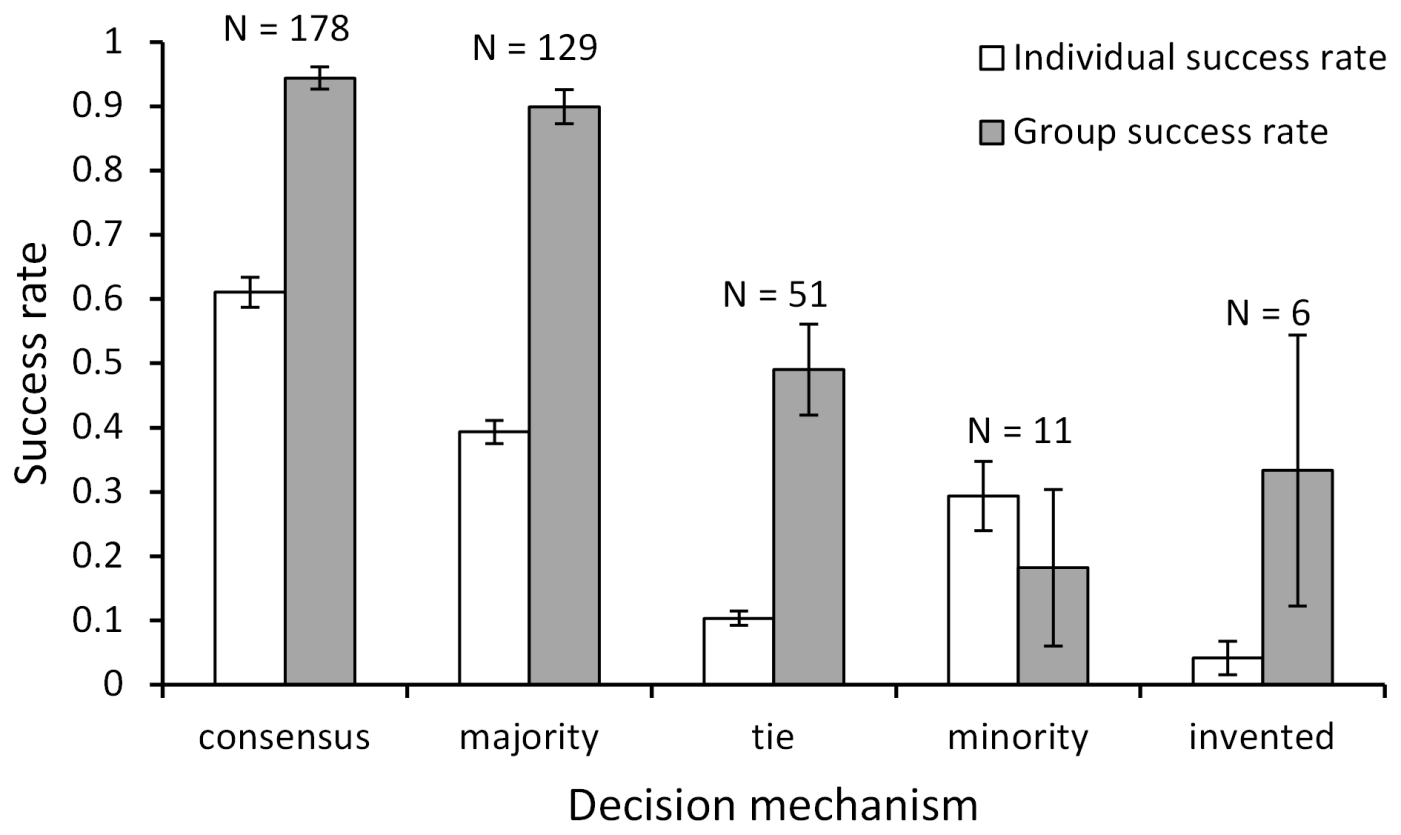
Mechanisms of group decisions. Shown are the different categories of how groups decided on a word based on the collection of independent responses/words. Consensus: all independent responses were identical to the group response; majority: the group response corresponded to the word that was most often reconstructed during the independent responses; tie: the group response corresponded to one of two (or more) words that were most often reconstructed during the independent responses; minority: the group response was present in the independent responses but was not one of the words that were most often given in the independent responses; invented: the group response was not present in the independent responses. Per category, the frequency (labelled as “N”) and the success rate (mean ± SE) of individuals (white bars) and groups (dark bars) are shown. Whenever an individual did not fill in a word as an independent response this was considered as ‘incorrect’. Majority decisions resulted in higher success rate and were much more frequent than minority decisions, which did not improve success rate.

Most of the group decisions when choosing individual words were based on consensus (n = 178) or majority (n = 129), followed by ties (n = 51) and minority (n = 11). A few words (n = 6) were not present in the individual responses but were invented (Figure 2). The success rate of groups was higher than individual success rate in all categories, except during minority voting (Figure 2, see also Discussion).

 The combination of best individual responses was significantly better than the group response (WER: 0.16 ± 0.08, *P* < 0.01; F-measure: 0.86 ± 0.09, *P* < 0.01).

## Discussion

We show that groups were able to decrease the number of errors and increase the semantic value of reconstructed sentences in a realistic context. In the individual treatment, extra time did not improve the performance, whereas in the group treatment the group outcome was better than the single best individual [21,22].

Groups performed better than their best individual both at the syntactic level (WER) and at the semantic level (F-measure). The developments in machine speech recognition and collective cognition have so far been separate fields of research. We believe that tools developed in machine speech recognition can open up new possibilities to study how language is used and processed by human groups. This can increase our understanding of how and why human groups use language. This is an important consideration, since language is thought to have played a critical role in the evolution of hominids [17] giving them a unique way of sharing information. Due to our limited number of groups (21) and sentences (2) further studies are, however, necessary to evaluate the robustness of our findings. An important consideration is how group improvement is affected by the complexity of the sentence in terms of syntax and semantics. Does group improvement occur only at a narrow range of complexity, or at a broad spectrum of complexity levels? Also further research is warranted to understand how group improvement in sentence reconstruction tasks scales with group size (see also 23).

In many previous studies on CC in humans, information is aggregated computationally by the experimenter, post-hoc [24-26] but see 27. Here we obtained independent information from the study subjects, who were then allowed to communicate. The aggregation was thus done by the subjects, simulating real group decision-making in humans. This communication is a key ingredient for CC to arise. It allows participants to exchange not only their opinions but also their level of confidence, a critical piece of information [28] enabling others to judge how relevant the separate pieces of information are. This allows groups to make better decisions than individuals even in the absence of feedback on individual performances [28]. It would be interesting to see how well groups would do in the absence of communication and only show individuals the opinions of their group members. This would allow quantification of the importance of the communication aspect. For simple tasks such as estimating quantities, and provided that estimations are independent and then aggregated, group size is one of the main predictors of decision accuracy [25]. However, for more complex tasks (such as sentence reconstructions) a benefit of CC with increasing group size is not always a given since larger groups might face communication difficulties. Moreover, CC is not suitable for all types of problems and in some cases it is better to follow the expert [21,25]. Group discussions can even impair decision accuracy due to the inequality of individual influence [29] because the opinion of others can negatively influence individual decisions [26].

Majority decisions occurred much more often than minority decisions (Figure 2) suggesting that there were no strong leaders or dominant individuals present that managed to override majorities [30]. During the group discussions, there was often a substantial proportion of individuals (on average 42.3%) that did not fill in a word which might have facilitated majority decisions since the presence of uninformed individuals can increase democratic, majority decisions [31]. Majority decisions led to much better decisions, as opposed to minority decisions which deteriorated decisions, illustrating that majority decision is a successful strategy in sentence reconstruction tasks. Most other types of decisions also led to better decisions (Figure 2) and in a few cases groups managed to find the correct word whereas it was not present in their individual responses. This, however, occurred only rarely and we did not find evidence in favour of the assembly bonus effect, which means that group performance is better than the performance of any individual group member or any combination of individual member efforts [19,20]. In contrast, the best possible combination of individual responses was significantly better than the group response indicating that although correct words were available, the groups were not always able to incorporate them into their final answer.

Ultimately, the critical test of collective cognition is the actual decision that groups and individuals would make (i.e., would they have caught the train or plane?). We did not study decision accuracy directly but extracted meaning from reconstructed sentences. Evaluating the pragmatic level is a difficult theoretical problem to solve [18]. One possibility is to ask people to carry out the task. However, if it is an everyday problem, people might not only use collective cognition but start using other strategies as well, such as looking at a map or asking professionals. And if on the other hand the problem is too limited and artificial, then the result would not be representative.

From an evolutionary point of view, the fact that groups beat even their best members shows that not only the average individuals, but also the top ones have an incentive to join a group to solve complex problems. Assessing the costs and benefits (to arrive at fitness measures) of such strategies (i.e. solving a problem alone or as part of a group) remains an important challenge for future studies.

## Supporting Information

Analysis S1 **Code for extracting the F-measure of the train station announcement.**
(PL) Click here for additional data file.

Analysis S2 **Code for extracting the F-measure of the airport announcement.**
(PL) Click here for additional data file.

Data S1 **F-measure and WER of all individuals, average individuals, best individuals and groups.**
(XLSX)
